# Supplementary material for: Changes to Urinary Proteome in High-Fat-Diet ApoE−/− Mice
Source: Biomolecules. 2022 Oct 26;12(11):1569. doi: 10.3390/biom12111569 (PMC9687344; doi:10.3390/biom12111569)
Supplement: Supplementary file 1 [file biomolecules-12-01569-s001.zip › biomolecules-1925924-supplementary.pdf]

Supplementary Material

Table S1. Screening results of random combinations of urine samples.

Group	Total number of random combinations	Average numbers of proteins with false random combinations	Number of correctly identified differential proteins	Percentage
EW1-EW0	231	8.47	27	31.37%
EM1-EW0	231	10.35	51	20.29%
EM2-EW0	231	9.99	69	14.48%
EM3-EW0	231	9.69	86	11.27%
EM4-EW0	231	8.67	65	13.33%
EM5-EW0	231	9.64	88	10.95%
EW0-CW0	126	9.66	54	17.89%
EW1-CW1	210	9.07	17	53.35%
EM1-CM1	210	8.31	55	15.11%
EM2-CM2	210	10.16	25	40.64%
EM3-CM3	210	9.24	58	15.93%
EM4-CM4	210	9.48	62	15.29%
EM5-CM5	210	8.53	55	15.51%

To test the influence of the possibility of random generation of different proteins on the screening results, every two groups of samples were randomly arranged and combined, and the average number of different proteins in all combinations was calculated according to the same screening conditions (Fc ≥1.5 or Fc ≤0.67, p <0.05). The average number of differential proteins produced by random grouping was significantly different from the number of differential proteins obtained by normal grouping screening, indicating that the differential proteins were indeed generated by the differences in samples within the group, which was relatively reliable.

Table S2. Differential proteins between week 1 and week 0 samples in the control group.

UniProt ID	Protein Name	P-value	Fold Change
	Calumenin		
O35887	Voltage-dependent anion-selective channel protein 1	0.0386	2.52
Q60932	Ig kappa chain V-III region PC 3741/TEPC 111	0.0382	1.82
P01660	Resistin-like gamma	0.0458	1.64
Q8K426	H-2 class I histocompatibility antigen, Q10 alpha	0.0119	1.59
P01898	chain	0.0290	0.67
P23953	Carboxylesterase 1C	0.0333	0.61
Q60847	Collagen alpha-1(XII) chain	0.0437	0.56
Q07456	Protein AMBP	0.0068	0.51
O88322	Nidogen-2	0.0063	0.51
Q9JJS0	Signal peptide, CUB and EGF-like domain-contain-	0.0135	0.46
P09036	ing protein 2	0.0443	0.44
	Serine protease inhibitor Kazal-type 1		

Table S3. Details of continuously changing differential proteins in the comparison within the experimental group.

UniProt	Human UniProt	Protein Name	Fold Change						References
			EW0	EM1	EM2	EM3	EM4	EM5	
B5X0G2	No	Major urinary protein 17	1	11.94	27.92	27.91	20.90	11.18	[20]
P11588	No	Major urinary protein 1	1	9.59	18.11	17.19	21.96	9.24	[20]
P01665	No	Ig kappa chain V-III region	1	—	11.01	8.41	18.15	9.95	—
Q61646	P00738	PC 7043	1	—	7.68	8.66	5.76	11.25	[30]

UniProt	Human UniProt	Protein Name	Fold Change						References
			EW0	EM1	EM2	EM3	EM4	EM5	
P04441	P04233	Haptoglobin	1	—	4.99	5.50	10.95	4.80	[31]
Q5FW60	No	H-2 class II histocompatibil-	1	4.24	6.10	5.57	7.53	4.28	[20]
P11591	No	ity antigen gamma chain	1	3.94	5.26	4.40	6.76	3.95	[20]
P0CW03	No	Major urinary protein 20	1	2.91	4.45	2.95	6.45	2.59	—
Q03404	Q03403	Major urinary protein 5	1	1.76	3.04	2.16	5.91	2.41	[21]
Q9Z0M9	O95998	Lymphocyte antigen 6C2	1	—	3.01	2.08	3.93	3.08	[32]
P11589	No	Trefoil factor 2	1	—	2.52	2.56	4.30	2.43	[20]
P17047	P13473	Interleukin-18-binding pro-	1	—	2.10	3.13	3.20	2.64	[33]
Q9Z0K8	O95497	tein	1	—	1.73	1.79	1.75	5.22	—
P09803	P12830	Major urinary protein 2	1	—	2.20	2.88	2.28	2.13	[34]
P61110	P61109	Lysosome-associated mem-	1	—	1.84	1.92	3.54	1.63	[23]
		brane glycoprotein 2							—
P01837	P01834	Pantetheinase	1	—	1.94	2.78	2.35	1.61	—
P01631	No	Cadherin-1	1	—	1.74	1.78	2.77	1.85	—
Q8R242	Q01459	Kidney androgen-regulated	1	—	1.65	2.16	1.99	2.08	[35]
P11276	P02751	protein	1	—	0.63	0.48	0.59	0.40	[36]
P07309	P02766		1	—	0.51	0.53	0.56	0.49	[37]
Q921I1	P02787	Immunoglobulin kappa	1	—	0.46	0.45	0.37	0.45	[22]
P11859	P01019	constant	1	0.44	0.47	0.46	0.37	0.35	[38]
P07759	P01011	Ig kappa chain V-II region	1	—	0.38	0.38	0.35	0.37	[23]
P07758	P01009	26-10	1	0.51	0.29	0.29	0.30	0.41	[24]
		Di-N-acetylchitobiase							[25]
O54775	O95388	Fibronectin	1	0.24	0.30	0.40	0.42	0.20	—
O88968	P20062	Transthyretin	1	0.36	0.37	0.27	0.31	0.19	[26]
Q4KML4	Q9P1F3	Serotransferrin	1	0.32	0.29	0.37	0.26	0.25	[39]
P35230	Q06141	Angiotensinogen	1	0.18	0.27	0.25	0.37	0.32	[27]
P02816	P12273	Serine protease inhibitor	1	—	0.30	0.24	0.21	0.35	[28]
P29699	P02765	A3K	1	0.39	0.27	0.16	0.32	0.11	[29]
P13020	P06396	Alpha-1-antitrypsin 1-1	1	0.39	0.27	0.18	0.24	0.15	—
Q9JJS0	Q9NQ36		1	0.23	0.24	0.17	0.23	0.09	—
O88322	Q14112	CCN family member 4	1	0.24	0.20	0.14	0.29	0.08	[23]
Q8C6C9	Q6P5S2	Transcobalamin-2	1	—	0.16	0.11	0.08	0.11	
P11087	P02452	Costars family protein	1	0.07	0.11	0.10	0.14	0.06	
		ABRACL							
		Regenerating islet-derived							
		protein 3-beta							
		Prolactin-inducible protein							
		homolog							
		Alpha-2-HS-glycoprotein							
		Gelsolin							
		Signal peptide, CUB and							
		EGF-like domain-containing							
		protein 2							
		Nidogen-2							
		Protein LEG1 homolog							
		Collagen alpha-1(I) chain							

Table S4. Differential proteins between adjacent time points of the experimental group.

Group	UniProt	Human UniProt	Protein Name	P-value	Fold Change
EM2 - EM1	P10287	P22223	Cadherin-3	0.0245	2.74
	Q07797	Q08380	Galectin-3-binding protein	0.0179	2.57
	P23780	P16278	Beta-galactosidase	0.0468	2.05
	Q8K1H9	Q9NY56	Odorant-binding protein 2a	0.0328	2.04
	Q9WVJ3	Q9Y646	Carboxypeptidase Q	0.0235	1.88
	Q91X17	P07911	Uromodulin	0.0074	1.86
	P55288	P55287	Cadherin-11	0.0395	1.80
	Q03404	Q03403	Trefoil factor 2	0.0131	1.73
	Q91WR6	Q9NU53	Glycoprotein integral membrane protein 1	0.0126	1.63
	Q9ESY9	P13284	Gamma-interferon-inducible lysosomal thiol reductase	0.0112	1.58
	P06909	P08603	Complement factor H	0.0192	0.61
	Q8BND5	O00391	Sulfhydryl oxidase 1	0.0176	0.55
	Q8K0E8	P02675	Fibrinogen beta chain	0.0329	0.24
EM3 - EM2	Q00993	P30530	Tyrosine-protein kinase receptor UFO	0.0114	2.05
	Q62267	No	Cornifin-B	0.0153	1.89
	P70663	Q14515	SPARC-like protein 1	0.0128	1.79
	P70699	P10253	Lysosomal alpha-glucosidase	0.0364	1.68
	P03953	P00746	Complement factor D	0.0176	0.64
	Q00898	P01009	Alpha-1-antitrypsin 1-5	0.0487	0.64
	Q9Z0L8	Q92820	Gamma-glutamyl hydrolase	0.0456	0.54
	Q60847	Q99715	Collagen alpha-1(XII) chain	0.0499	0.51
	Q9EP95	Q9BQ08	Resistin-like alpha	0.0201	0.26
EM4 - EM3	P25119	P20333	Tumour necrosis factor receptor superfamily member 1B	0.0491	3.19
	Q03404	Q03403	Trefoil factor 2	0.0237	2.74
	Q9DAU7	Q14508	WAP four-disulfide core domain protein 2	0.0293	2.61
	Q9EQC7	O95633	Follistatin-related protein 3	0.0460	2.58
	Q07797	Q08380	Galectin-3-binding protein	0.0410	2.57
	Q61581	Q16270	Insulin-like growth factor-binding protein 7	0.0404	2.50
	Q01339	P02749	Beta-2-glycoprotein 1	0.0411	2.45
	Q6SJQ5	Q6UXZ3	CMRF35-like molecule 3	0.0430	2.28
	P15379	P16070	CD44 antigen	0.0313	2.18
	Q91WR8	P59796	Glutathione peroxidase 6	0.0096	1.99
	Q9Z0M9	O95998	Interleukin-18-binding protein	0.0294	1.89
	Q5SSE9	Q86UQ4	ATP-binding cassette sub-family A member 13	0.0279	1.69
	P01132	P01133	Pro-epidermal growth factor	0.0125	1.69
	P01631	No	Ig kappa chain V-II region 26-10	0.0087	1.56
	P07724	P02768	Albumin	0.0193	0.57
EM5 - EM4	P01864	No	Ig gamma-2A chain C region secreted form	0.0121	3.35
	Q9ET22	Q9UHL4	Dipeptidyl peptidase 2	0.0002	3.31
	Q9Z0K8	O95497	Pantetheinase	0.0160	2.98
	P00688	P04746	Pancreatic alpha-amylase	0.0072	2.76
	P13634	P00915	Carbonic anhydrase 1	0.0017	2.51
	A2ARV4	P98164	Low-density lipoprotein receptor-related protein 2	0.0248	2.11
	P51910	P05090	Apolipoprotein D	0.0496	2.04
	Q9WUU7	Q9UBR2	Cathepsin Z	0.0121	1.78
	P16675	P10619	Lysosomal protective protein	0.0190	1.77
	P07724	P02768	Albumin	0.0094	1.68
	Q06890	P10909	Clusterin	0.0384	1.52
	Q61147	P00450	Ceruloplasmin	0.0491	1.52

Group	UniProt	Human UniProt	Protein Name	P-value	Fold Change
	P01631	No	Ig kappa chain V-II region 26-10	0.0067	0.67
	P61110	P61109	Kidney androgen-regulated protein	0.0270	0.46
	Q61129	P05156	Complement factor I	0.0213	0.44
	P04441	P04233	H-2 class II histocompatibility antigen gamma chain	0.0483	0.44
	Q01339	P02749	Beta-2-glycoprotein 1	0.0439	0.42
	P42567	P42566	Epidermal growth factor receptor substrate 15	0.0482	0.41
	Q03404	Q03403	Trefoil factor 2	0.0317	0.41
	Q91WR8	P59796	Glutathione peroxidase 6	0.0021	0.38
	Q61271	P36896	Activin receptor type-1B	0.0275	0.35
	Q62395	Q07654	Trefoil factor 3	0.0402	0.34
	P29699	P02765	Alpha-2-HS-glycoprotein	0.0484	0.34
	Q08423	P04155	Trefoil factor 1	0.0447	0.32
	P10287	P22223	Cadherin-3	0.0126	0.31
	Q9EP95	Q9BQ08	Resistin-like alpha	0.0479	0.30
	O08997	O00244	Copper transport protein ATOX1	0.0378	0.28
	P0CG49	P0CG47	Polyubiquitin-B	0.0095	0.28
	O88322	Q14112	Nidogen-2	0.0196	0.28
	Q7TMJ8	Q96FE7	Phosphoinositide-3-kinase-interacting protein 1	0.0360	0.26
	O09051	Q16661	Guanylate cyclase activator 2B	0.0252	0.23
	P09036	P00995	Serine protease inhibitor Kazal-type 1	0.0200	0.19
	Q62267	No	Cornifin-B	0.0500	0.19

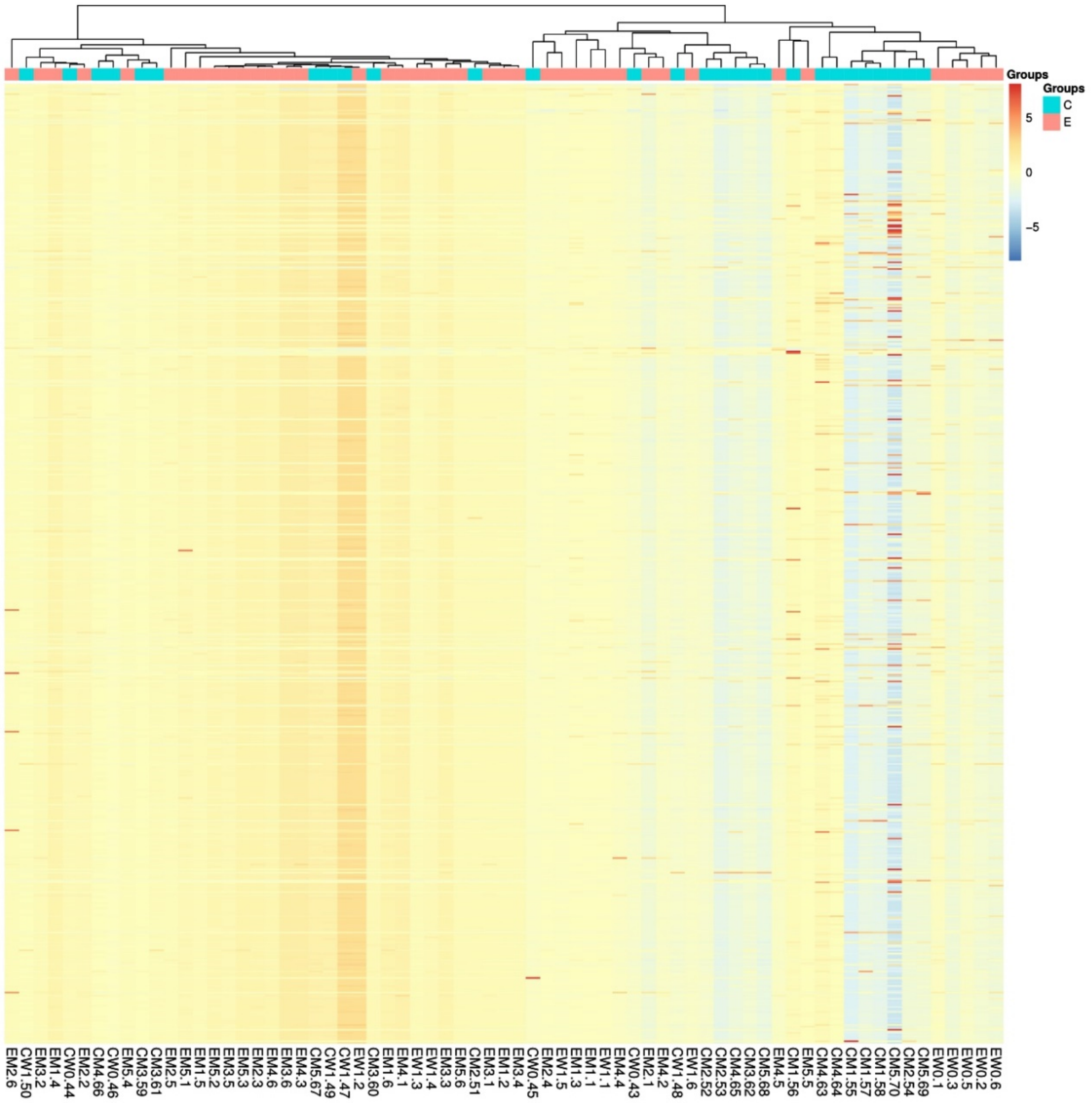

Figure S1. Venn diagram of differential proteins at different time points between the experimental group and the control group.

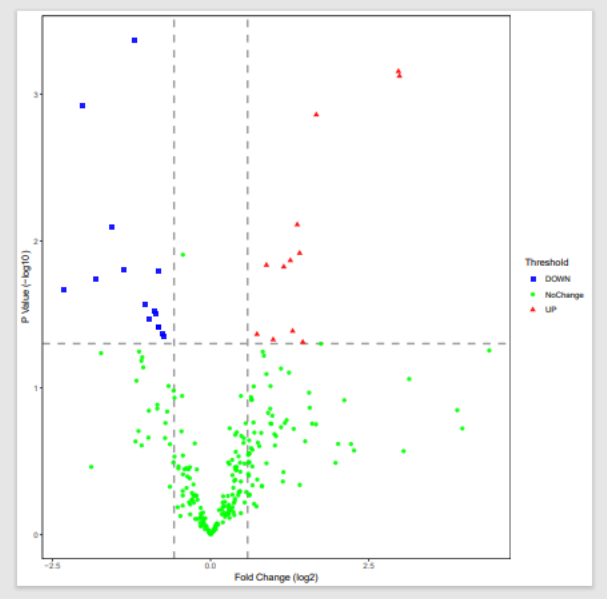

EW1-EW0

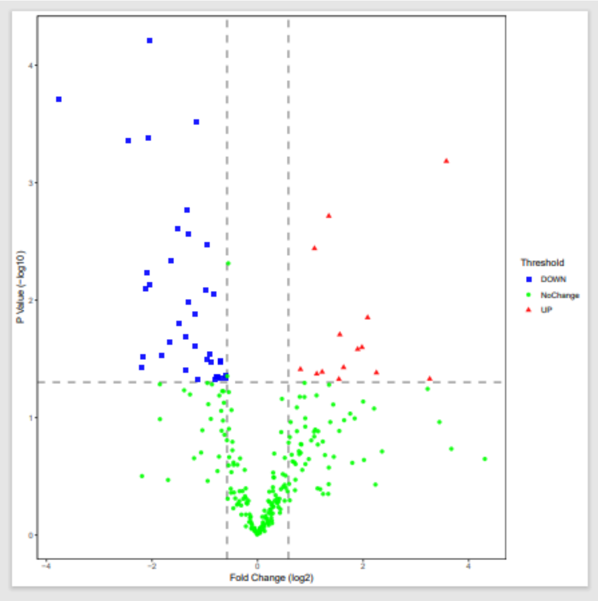

EM1-EW0

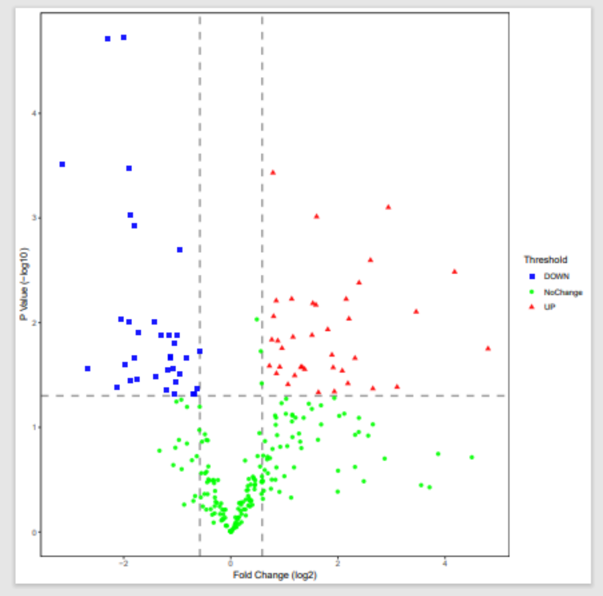

EM2-EW0

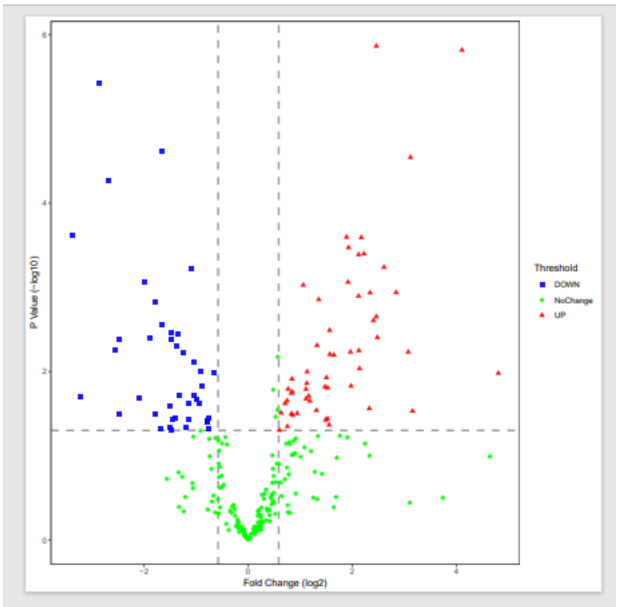

EM3-EW0

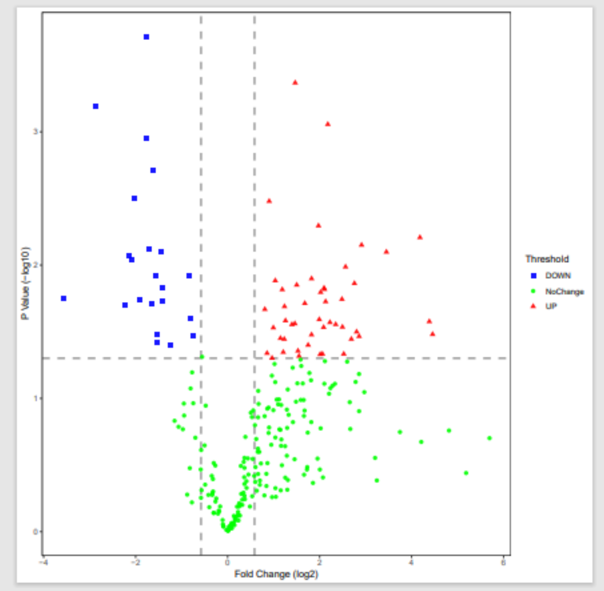

EM4-EW0

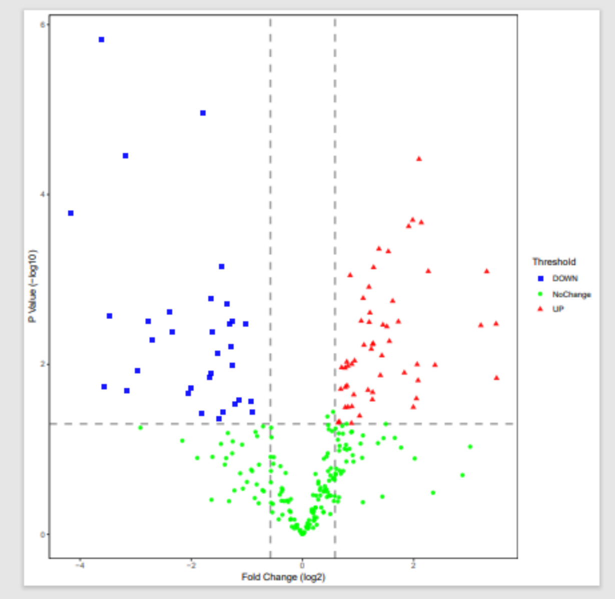

EM5-EW0

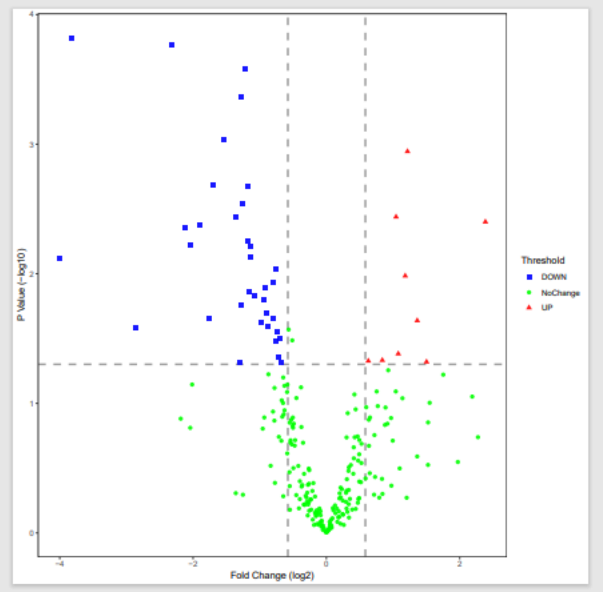

EW0-CW0

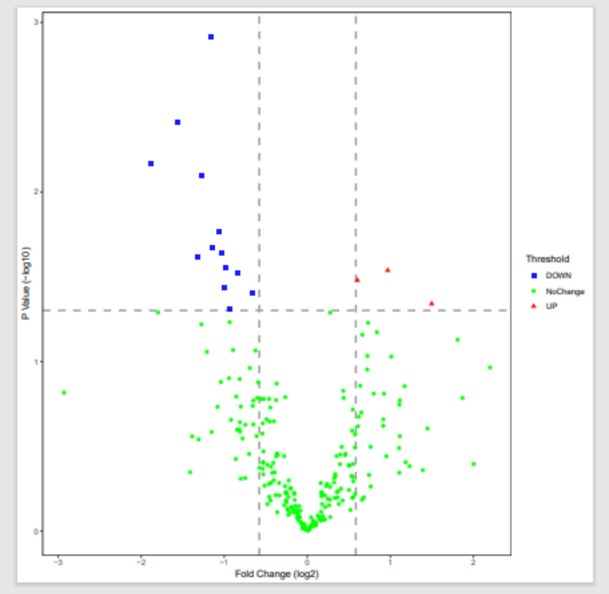

EW1-CW1

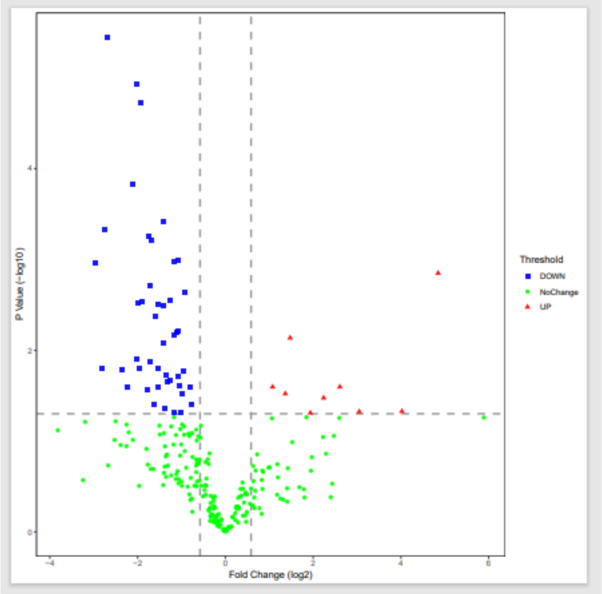

EM1-CM1

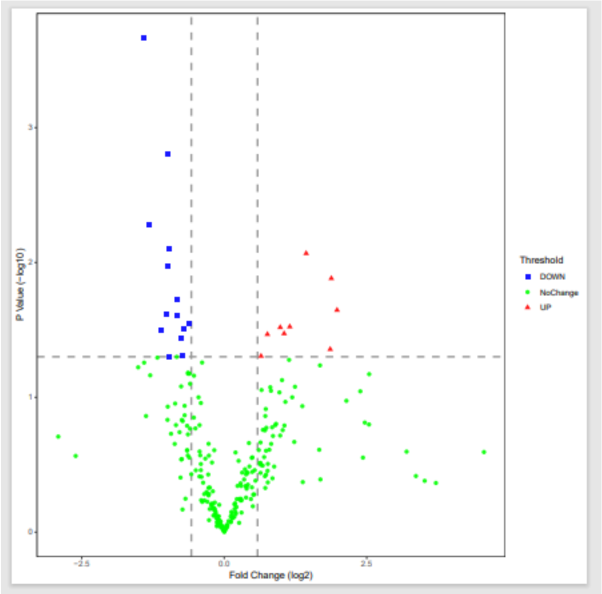

EM2-CM2

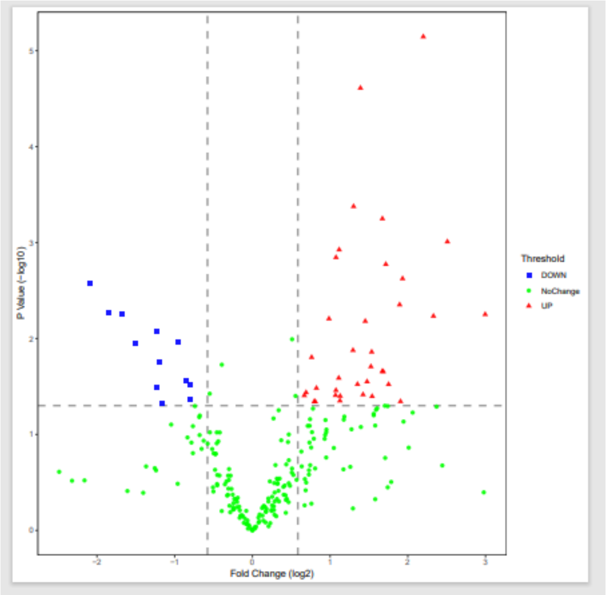

EM3-CM3

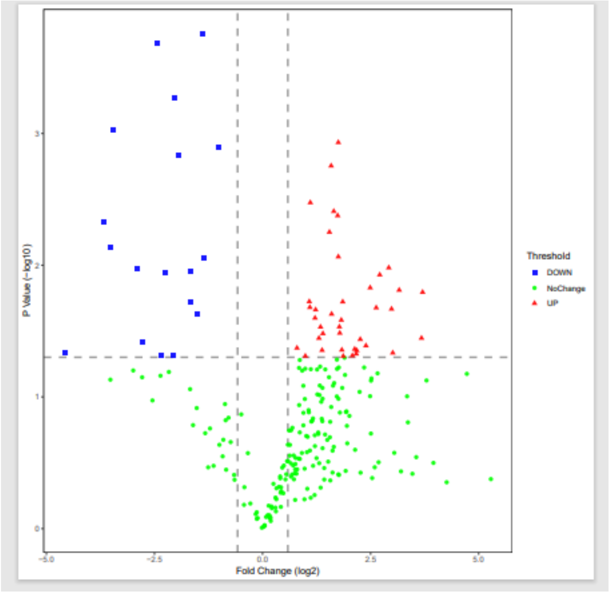

EM4-CM4

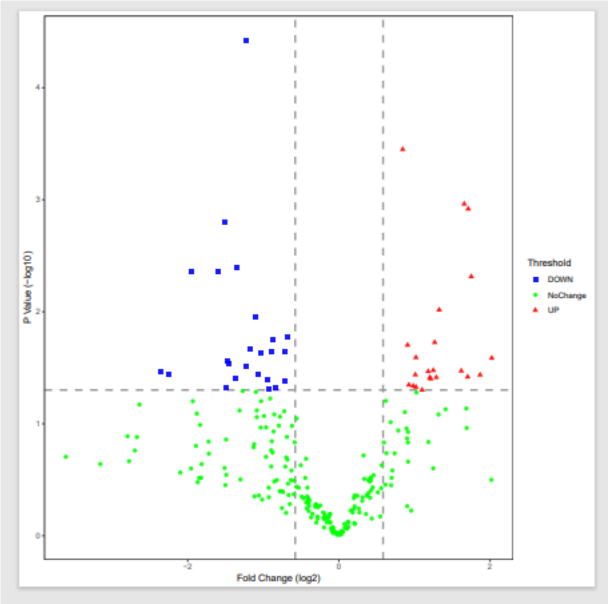

EM5-CM5

Figure S2. Volcano plots of proteins.

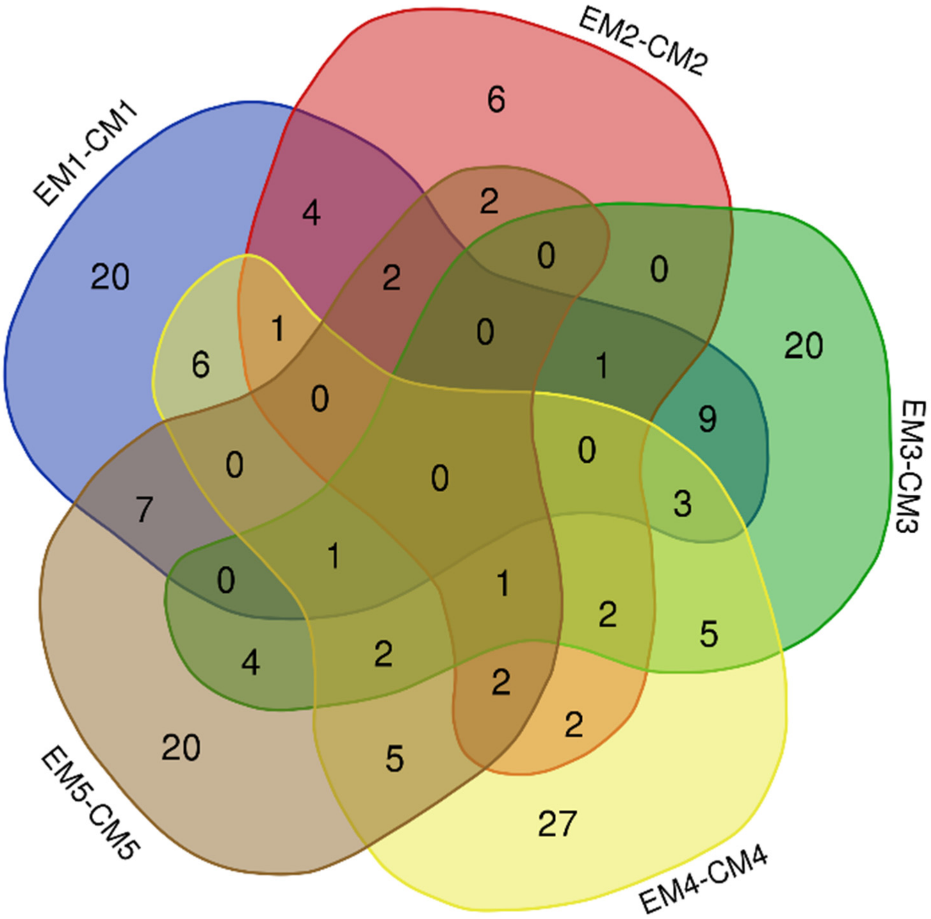

Figure S3. Venn diagram of differential proteins at different time points between the experimental group and the control group.

Table S5. Details of differential modifications in two comparisons by open search.

Group	Modification Name	Modification Type	P-value	Fold Change
	Oxidation[P]	Post-translational	①	①
	4-ONE[H]	Chemical derivative	②	②
	AccQTag[AnyN-term]	Chemical derivative	②	②
	AEBS[K]	Artefact	②	②
	Cation_K[D]	Artefact	②	②
	Delta_H(2)C(3)[K]	Other	②	②
	Ethyl+Deamidated[N]	Chemical derivative	②	②
	Hep[T]	O-linked glycosylation	②	②
	NHS-LC-Biotin[AnyN-term]	Chemical derivative	②	②
	NO_SMX_SIMD[C]	Chemical derivative	②	②
	Arg->Ser[R]	AA substitution	②	②
	CHDH[D]	Post-translational	②	②
	Diisopropylphosphate[AnyN-term]	Chemical derivative	②	②
	glucosone[R]	Other	②	②
	Gly->His[G]	AA substitution	②	②
	Hex[K]	Other glycosylation	②	②
	ICDID[C]	Artefact	②	②
	ICPL_2H(4)[AnyN-term]	Artefact	②	②
	LG-anhydrolactam[AnyN-term]	Post-translational	②	②
	NDA[K]	Chemical derivative	②	②
	Phe->Gly[F]	AA substitution	②	②
	Phospho[C]	Post-translational	②	②
EM5	Phosphoadenosine[T]	Post-translational	②	②
-	SMA[AnyN-term]	Chemical derivative	②	②
EW0	BITC[AnyN-term]	Chemical derivative	②	②
	Bromo[F]	Post-translational	②	②
	Cytopiloyne+water[AnyN-term]	Chemical derivative	②	②
	DAET[S]	Chemical derivative	②	②
	Delta_Hg(1)[C]	Chemical derivative	②	②
	Didehydro[Y]	Post-translational	②	②
	Dimethylphosphothione[C]	Chemical derivative	②	②
	Glu->Tyr[E]	AA substitution	②	②
	Hex(2)[T]	O-linked glycosylation	②	②
	HexNAc(2)Sulf(1)[S]	O-linked glycosylation	②	②
	HNE+Delta_H(2)[K]	Chemical derivative	②	②
	Iminobiotin[AnyN-term]	Chemical derivative	②	②
	Iodoacetanilide_13C(6)[AnyN-term]	Artefact	②	②
	Nitro[F]	Artefact	②	②
	Oxidation[V]	Chemical derivative	②	②
	Oxidation+NEM[C]	Chemical derivative	②	②
			②	②
			②	②
			②	②
			②	②
			②	②
	PhosphoUridine[Y]	Post-translational	28.64	0.0378
	Thiophospho[T]	Other	21.41	0.0151
	Xle->Phe[I]	AA substitution	14.55	0.0002
	Xlink_SMCC[219][C]	Chemical derivative	14.17	0.0194
	Xlink_SMCC[237][C]	Chemical derivative	13.45	0.0082
			13.24	0.0000
	NEIAA[C]	Artefact	13.08	0.0009
	ICPL_13C(6)[AnyN-term]	Artefact	12.72	0.0348

Xle->Trp[I]	AA substitution	12.15	0.0007
Thiazolidine[W]	Chemical derivative	11.97	0.0013
SMA[K]	Chemical derivative	10.05	0.0106
NEMsulfurWater[C]	Chemical derivative	9.40	0.0035
Guanidinyl[K]	Chemical derivative	8.81	0.0002
Phosphopropargyl[S]	Multiple	7.43	0.0086
Thiophospho[S]	Other	7.42	0.0247
phenylsulfonylethyl[C]	Chemical derivative	7.11	0.0250
Ub-Br2[C]	Chemical derivative	6.62	0.0200
Asn->Trp[N]	AA substitution	6.39	0.0015
NEMsulfur[C]	Chemical derivative	6.26	0.0012
Carboxyethyl[K]	Post-translational	6.07	0.0070
Ethanedithiol[T]	Chemical derivative	5.80	0.0025
Met->Glu[M]	AA substitution	5.18	0.0054
DeStreak[C]	Chemical derivative	5.13	0.0316
Xlink_EGS[115][K]	Chemical derivative	4.64	0.0410
Xle->Tyr[L]	AA substitution	4.58	0.0379
2-succinyl[C]	Chemical derivative	4.47	0.0046
Cys->SecNEM_2H(5)[C]	Chemical derivative	4.42	0.0341
Phenylisocyanate_2H(5)[AnyN-term]	Chemical derivative	4.35	0.0222
Asn->His[N]	AA substitution	4.13	0.0371
SulfurDioxide[C]	Post-translational	4.07	0.0054
Piperidine[AnyN-term]	Chemical derivative	3.97	0.0065
Tris[N]	Artefact	3.88	0.0410
Delta_H(-4)O(2)[W]	Chemical derivative	3.72	0.0001
Asn->Cys[N]	AA substitution	3.72	0.0039
Carboxymethyl[AnyN-term]	Artefact	3.63	0.0468
Xle->Tyr[I]	AA substitution	3.56	0.0113
Propionamide[C]	Artefact	3.55	0.0012
Xle->Cys[I]	AA substitution	3.46	0.0499
Carboxymethyl[K]	Artefact	3.44	0.0059
Isopropylphospho[T]	Chemical derivative	3.43	0.0001
Cation_Ni[II][D]	Artefact	3.40	0.0094
Trp->Kynurenin[W]	Chemical derivative	3.34	0.0196
2-monomethylsuccinyl[C]	Chemical derivative	3.28	0.0016
PET[T]	Chemical derivative	3.26	0.0100
Cysteinyl[C]	Multiple	3.10	0.0481
Nmethylmaleimide+water[C]	Chemical derivative	2.87	0.0318
MDCC[C]	Chemical derivative	2.86	0.0036
MercaptoEthanol[T]	Chemical derivative	2.85	0.0399
Acetyl[AnyN-term]	Multiple	2.81	0.0195
Formylasparagine[H]	Chemical derivative	2.75	0.0008
Glucuronyl[T]	O-linked glycosylation	2.65	0.0437
O-Methylphosphate[S]	Chemical derivative	2.64	0.0114
Trioxidation[C]	Chemical derivative	2.49	0.0071
Propionamide[AnyN-term]	Chemical derivative	2.44	0.0268
Asn->Met[N]	AA substitution	2.32	0.0048
Carbamyl[AnyN-term]	Multiple	2.24	0.0444
Cation_Ni[II][E]	Artefact	2.14	0.0283
Cation_Fe[II][E]	Artefact	2.06	0.0037
Trp->Oxolactone[W]	Chemical derivative	1.96	0.0295
Arg[AnyN-term]	Other	1.84	0.0000
dHex[N]	N-linked glycosylation	1.76	0.0143

	Amidine[AnyN-term]	Chemical derivative	1.68	0.0161
	Carboxy[W]	Chemical derivative	1.55	0.0318
	Nmethylmaleimide[C]	Chemical derivative	0.45	0.0008
	Dethiomethyl[M]	Artefact	0.39	0.0011
	Unknown_210[AnyN-term]	Artefact	0.39	0.0087
	Gly->Pro[G]	AA substitution	0.36	0.0006
	Formyl[T](Thr->Glu[T])	Artefact	0.34	0.0000
	Xle->Gln[I]	AA substitution		
	CarbamidomethylDTT[C]	Artefact		
	Pyro-carbamidomethyl[AnyN-termC]	Artefact		
	Thr->Ala[T]	AA substitution		
	Deoxy[S](Ser->Ala[S])	Chemical derivative		
	Gln->pyro-Glu[AnyN-termQ]	Artefact		
	Arg->Trp[R]	AA substitution	③	③
	His->Ser[H]	AA substitution	③	③
	HexNAc(2)[N]	N-linked glycosylation	③	③
	HN2_mustard[C]	Post-translational	③	③
	Oxidation[P]	Post-translational	③	③
	Pro->His[P]	AA substitution	③	③
	Val->Glu[V]	AA substitution	③	③
	Xle->Val[I]	AA substitution	③	③
			②	②
	Delta_H(2)C(3)[K]	Other	②	②
	DeStreak[C]	Chemical derivative	②	②
	Dihydroxyimidazolidine[R]	Multiple	②	②
	Guanidinyl[K]	Chemical derivative	②	②
	phenylsulfonylethyl[C]	Chemical derivative	②	②
	Cation_Cu[I][E]	Artefact	②	②
	glucosone[R]	Other	②	②
	Hex[K]	Other glycosylation	②	②
	HexNAc(1)dHex(1)[T]	O-linked glycosylation	②	②
EM5	ICDID[C]	Artefact	②	②
-	ICPL_2H(4)[AnyN-term]	Artefact	②	②
CM5	LG-anhydrolactam[AnyN-term]	Post-translational	②	②
	mTRAQ[AnyN-term]	Artefact	②	②
	NDA[K]	Chemical derivative	②	②
	Phe->Gly[F]	AA substitution	②	②
	Quinone[W]	Post-translational	②	②
	Quinone[Y]	Post-translational	②	②
	SMA[K]	Chemical derivative	②	②
	Thiazolidine[F]	Chemical derivative	②	②
	2-hydroxyisobutyrylation[K]	Post-translational	②	②
	BITC[K]	Chemical derivative	②	②
	Bromo[F]	Post-translational	②	②
	Cytopiloyne+water[AnyN-term]	Chemical derivative	②	②
	DAET[S]	Chemical derivative	②	②
	Delta_Hg(1)[C]	Chemical derivative	②	②
	HexNAc(2)Sulf(1)[S]	O-linked glycosylation	②	②
	HNE+Delta_H(2)[K]	Chemical derivative	②	②
	Malonyl[C]	Chemical derivative	②	②
	MTSL[C]	Chemical derivative	②	②
	Oxidation+NEM[C]	Chemical derivative	②	②
			②	②
			②	②
			②	②
			②	②

Phe->Thr[F]	AA substitution	②	②
PhosphoUridine[Y]	Post-translational	②	②
Ser->Trp[S]	AA substitution		
SulfanilicAcid_13C(6)[E]	Chemical derivative	66.16	0.0010
Xle->Phe[I]	AA substitution	58.75	0.0142
Xlink_SMCC[219][C]	Chemical derivative	19.24	0.0155
Xlink_SMCC[237][C]	Chemical derivative	17.34	0.0251
		12.42	0.0436
Thiophospho[S]	Other	12.32	0.0436
NHS-LC-Biotin[AnyN-term]	Chemical derivative	11.08	0.0007
Ub-Br2[C]	Chemical derivative	10.95	0.0314
4-ONE[H]	Chemical derivative	10.75	0.0233
Lys->Pro[K]	AA substitution	9.42	0.0011
Xle->Thr[I]	AA substitution	8.68	0.0452
Acetyl[AnyN-term]	Multiple	8.63	0.0271
Oxidation[E]	Chemical derivative	8.04	0.0003
methylsulfonylethyl[C]	Chemical derivative	7.96	0.0316
O-Methylphosphate[S]	Chemical derivative	7.95	0.0069
SulfurDioxide[C]	Post-translational	7.37	0.0155
Phosphoadenosine[T]	Post-translational	7.15	0.0018
Carbamyl[AnyN-term]	Multiple	7.01	0.0018
Gly->His[G]	AA substitution	6.97	0.0025
Asn->Thr[N]	AA substitution	6.82	0.0000
Asn->Cys[N]	AA substitution	6.67	0.0012
Isopropylphospho[T]	Chemical derivative	6.58	0.0313
AccQTag[AnyN-term]	Chemical derivative	6.17	0.0019
Cys->SecNEM_2H(5)[C]	AA substitution	5.83	0.0148
Carboxymethyl[K]	Chemical derivative	5.59	0.0005
NEMsulfur[C]	Chemical derivative	5.48	0.0284
Ethyl+Deamidated[Q]	Chemical derivative	5.45	0.0249
Crotonaldehyde[C]	Other	5.36	0.0477
Hydroxymethyl[N]	Post-translational	5.33	0.0035
Gly->Pro[G]	AA substitution	5.32	0.0481
Acetyl_2H(3)[AnyN-term]	Artefact	5.27	0.0354
Carboxyethyl[K]	Post-translational	5.25	0.0000
Oxidation[Y]	Post-translational	5.20	0.0103
Cyano[C]	Post-translational	4.87	0.0075
Piperidine[AnyN-term]	Chemical derivative	4.68	0.0061
Cation_Ni[II][D]	Artefact	4.58	0.0032
Nmethylmaleimide+water[C]	Chemical derivative	4.14	0.0040
MercaptoEthanol[T]	Chemical derivative	4.11	0.0176
Carbonyl[I]	Chemical derivative	4.01	0.0002
Val->Trp[V]	AA substitution	3.65	0.0135
Xle->Trp[I]	AA substitution	3.51	0.0000
Propionyl[AnyN-term]	Artefact	3.48	0.0092
Amidine[AnyN-term]	Chemical derivative	3.47	0.0118
DNCB_hapten[C]	Chemical derivative	3.46	0.0347
Phenylisocyanate_2H(5)[AnyN-term]	Chemical derivative	3.42	0.0058
dHex[N]	N-linked glycosylation	3.20	0.0198
C+12[AnyN-term]	*	3.18	0.0000
GIST-Quat_2H(3)[AnyN-term]	Artefact	3.15	0.0011
Asn->Trp[N]	AA substitution	3.08	0.0275
Trioxidation[C]	Chemical derivative	3.05	0.0452

Dioxidation[M]	Post-translational	3.05	0.0007
Unknown_210[AnyN-term]	Artefact	3.03	0.0006
MG-H1[R](Delta_H(2)C(3)O(1)[R])	Other	3.02	0.0075
Tris[N]	Artefact	3.01	0.0245
Asn->Met[N]	AA substitution	2.87	0.0142
NEMsulfurWater[C]	Chemical derivative	2.75	0.0165
CarboxymethylDTT[C]	Artefact	2.74	0.0020
Cation_Fe[II][D]	Artefact	2.65	0.0134
Carboxy[W]	Chemical derivative	2.50	0.0354
Gly->Glu[G]	AA substitution	2.36	0.0403
Arg[AnyN-term]	Other	2.35	0.0472
Nmethylmaleimide[C]	Chemical derivative	2.35	0.0084
Oxidation[H]	Artefact	2.34	0.0309
Cation_K[D]	Artefact	2.30	0.0237
Cation_Fe[II][E]	Artefact	1.96	0.0178
Formylasparagine[H]	Chemical derivative	1.77	0.0105
Xle->Gln[I]	AA substitution	1.76	0.0190
Delta_H(2)C(2)[AnyN-term]	Other	1.73	0.0164
Cysteinyl[C]	Multiple	1.65	0.0066
Biotin_Thermo-88317[Y]	Chemical derivative	0.34	0.0401
Formyl[AnyN-term]	Artefact	0.29	0.0223
Xlink_EGS[115][K]	Chemical derivative	0.12	0.0037
Oxidation[W]	Artefact		
Cys->Dha[C]	AA substitution		
Gln->pyro-Glu[AnyN-termQ]	AA substitution		
dichlorination[Y]	Artefact		
azole[C]	Post-translational		

* Modification type not retrieved in UNIMOD database; ① The unique modification type of the EW0 group; ② The unique modification types of the EM5 group; ③ The unique modification types of the CM5 group.

Table S6. Results of limited search of modifications.

Group	Modification Name	Modification Type	P-value	Fold Change	References
EM5 - EW0	Carbamyl[AnyN-term]	Multiple	0.0000	4.65	[138]
	CHDH[D]	Post-translational	0.0005	2.31	—
	Trp->Kynurenin[W]	Chemical derivative	0.0375	2.04	[139]
	NHS-LC-Biotin[AnyN-term]	Chemical derivative	0.1206	1.92	—
	HNE+Delta_H(2)[K]	Chemical derivative	0.2029	1.69	[144]
	Thiazolidine[W]	Chemical derivative	0.9862	1.00	[147]
	Carboxyethyl[K]	Post-translational	0.7766	0.91	[148]
	PhosphoUridine[Y]	Post-translational	0.7787	0.90	[150]
	glucosone[R]	Other	0.6874	0.86	[145]
	Hep[T]	O-linked glycosylation	0.5316	0.83	—
	Ethyl+Deamidated[N]	Chemical derivative	0.1841	0.83	—
	dHex[N]	N-linked glycosylation	0.3045	0.74	[149]
	AccQTag[AnyN-term]	Chemical derivative	0.0364	0.70	—
	4-ONE[H]	Chemical derivative	0.2781	0.52	[145]
	Oxidation[P]	Post-translational	0.0000	0.47	[140]
	Cysteinyl[C]	Multiple	0.0234	0.43	[141]
	BITC[AnyN-term]	Chemical derivative	0.1039	0.39	[146]
	SulfurDioxide[C]	Post-translational	0.0050	0.33	[142]
	NO_SMX_SIMD[C]	Chemical derivative	0.0003	0.31	—
	Delta_H(2)C(3)[K]	Other	0.0045	0.29	[143]
EM5 - CM5	Guanidinyl[K]	Chemical derivative	0.0093	2.72	[156]
	Carboxymethyl[K]	Chemical derivative	0.1476	2.46	[155]
	PhosphoUridine[Y]	Post-translational	0.0204	1.88	[150]
	Carbamyl[AnyN-term]	Multiple	0.0061	1.76	[138]
	DeStreak[C]	Chemical derivative	0.2293	1.33	—
	glucosone[R]	Other	0.5228	1.32	[145]
	Crotonaldehyde[C]	Other	0.4124	1.30	[142]
	BITC[K]	Chemical derivative	0.5487	1.22	[146]
	Arg->Trp[R]	AA substitution	0.4018	1.21	—
	2-hydroxyisobutyrylation[K]	Post-translational	0.6458	1.15	[154]
	Cysteinyl[C]	Multiple	0.8368	1.09	[141]
	4-ONE[H]	Chemical derivative	0.8445	1.06	[144]
	dHex[N]	N-linked glycosylation	0.8163	1.05	[149]
	phenylsulfonylethyl[C]	Chemical derivative	0.9917	1.00	—
	Delta_H(2)C(3)[K]	Other	0.5922	0.93	[142]
	Oxidation[Y]	Post-translational	0.2648	0.86	[156]
	SulfurDioxide[C]	Post-translational	0.3743	0.80	[142]
	Malonyl[C]	Chemical derivative	0.3600	0.77	[152]
	HNE+Delta_H(2)[K]	Chemical derivative	0.5489	0.76	[144]
	MG-H1[R](Delta_H(2)C(3)O(1)[R])	Other	0.2428	0.72	[151]
	His->Ser[H]	AA substitution	0.1070	0.60	—
	Carboxyethyl[K]	Post-translational	0.2808	0.53	[148]
	Oxidation[P]	Post-translational	0.1375	0.47	[140]
	Delta_H(2)C(2)[AnyN-term]	Other	0.0260	0.39	[151]
	Dihydroxyimidazolidine[R]	Multiple	0.0147	0.31	—
